# Polymer Microparticles Prolong Delivery of the 15-PGDH Inhibitor SW033291

**DOI:** 10.3390/pharmaceutics14010085

**Published:** 2021-12-30

**Authors:** Alan B. Dogan, Nathan A. Rohner, Julianne N. P. Smith, Jessica A. Kilgore, Noelle S. Williams, Sanford D. Markowitz, Horst A. von Recum, Amar B. Desai

**Affiliations:** 1 Department of Biomedical Engineering, Case Western Reserve University, Cleveland, OH 44106, USA; Abd51@case.edu (A.B.D.); nathanrohner@gmail.com (N.A.R.); hav1@case.edu (H.A.v.R.); 2 Department of Medicine and Case Comprehensive Cancer Center, Case Western Reserve University, Cleveland, OH 44106, USA; Julianne.noel@gmail.com (J.N.P.S.); sxm10@case.edu (S.D.M.); 3 Department of Biochemistry, UT Southwestern Medical Center, Dallas, TX 75390, USA; Jessica.Kilgore@utsouthwestern.edu (J.A.K.); Noelle.Williams@utsouthwestern.edu (N.S.W.); 4 University Hospitals Seidman Cancer Center, Cleveland, OH 44106, USA

**Keywords:** fibrosis, 15-PGDH, SW033291, cyclodextrin, affinity

## Abstract

As the prevalence of age-related fibrotic diseases continues to increase, novel antifibrotic therapies are emerging to address clinical needs. However, many novel therapeutics for managing chronic fibrosis are small-molecule drugs that require frequent dosing to attain effective concentrations. Although bolus parenteral administrations have become standard clinical practice, an extended delivery platform would achieve steady-state concentrations over a longer time period with fewer administrations. This study lays the foundation for the development of a sustained release platform for the delivery of (+)SW033291, a potent, small-molecule inhibitor of the 15-hydroxyprostaglandin dehydrogenase (15-PGDH) enzyme, which has previously demonstrated efficacy in a murine model of pulmonary fibrosis. Herein, we leverage fine-tuned cyclodextrin microparticles—specifically, β-CD microparticles (β-CD MPs)—to extend the delivery of the 15-PGDH inhibitor, (+)SW033291, to over one week.

## 1. Introduction

In a normal wound healing response, fibroblast activity is essential for remodeling the extracellular matrix (ECM) after injury. However, in instances of unbalanced or uncontrolled tissue remodeling, the excessive deposition of ECM components results in fibrosis, which is a common pathological outcome of several chronic inflammatory diseases [1]. During fibrosis, connective tissue replaces parenchymal tissue, rendering the inflicted tissue partially or completely inflamed or damaged. Clinically, the most common manifestations of fibrotic damage can be seen in chronic diseases such as end-stage liver failure, kidney disease, heart failure, and idiopathic pulmonary fibrosis (IPF) [2].

Markedly, IPF, a chronic interstitial lung disease characterized by dyspnea, cough and increasing immobility, is particularly prevalent in 60–75-year-old patients with a history of smoking and/or occupational exposure to inhaled hazards [3]. Although the disease is expected to increase in prevalence in an increasingly aging population, current treatment options for IPF only extend the average life to 9–11 years after diagnosis [4,5]. To date, two drugs have been clinically approved for IPF—pirfenidone and nintedanib. However, both small-molecule drugs suffer from short half-lives, adverse drug reactions, high costs, and frequent oral dosing (TID, 3× daily and BID, 2× daily, respectively) [6,7]. Additionally, a large percentage of elderly patients discontinue taking these drugs due to gastrointestinal and other off-target complications [8].

Inhibition of 15-PGDH, the primary enzyme responsible for Prostaglandin E2 metabolism, using the small molecule (+)SW033291, has recently demonstrated preclinical efficacy in a murine model of bleomycin-induced IPF by limiting systemic inflammatory load and reducing pulmonary collagen deposition [9,10]. Further preclinical optimization would involve ensuring that (+)SW033291 can be effectively delivered to the region of treatment with minimized off-target action. Therefore, we propose to expand the clinical utility of 15-PGDH inhibition (PGDHi) by developing a delivery system to administer sustained PGDHi in chronic disease such as IPF. We predict that sustained 15-PGDH inhibition via injectable, cyclodextrin-derived microparticles will be a novel tolerated strategy to not only reduce fibrotic deposition and decrease morbidity and mortality in murine pulmonary fibrosis, but to also reduce the demands on the patient to comply with therapeutic dosing regimens, with clinical implications for a number of additional fibrotic conditions.

To this end, we propose the use of crosslinked cyclodextrin microparticles (CD MPs) as a vehicle for the sustained release of the 15-PGDH inhibitor (+)SW033291. CD systems have previously been shown to enhance the solubility and bioavailability of drugs by complexing small-molecule, hydrophobic payloads within its interior pocket, forming an ‘inclusion complex’ [11]. Furthermore, CD systems with high densities of neighboring inclusion complexes are able to leverage their thermodynamic interactions with the payload to yield a controlled-release delivery system that can deliver drug for 28–70 days [12,13,14,15,16]. Our group has also shown that implanted cyclodextrin-based systems have the potential to ‘refill’ after being exposed to a second bolus dose in situ, which would present the opportunity for an adjustable and tunable window of drug delivery, as opposed to similar polymeric delivery systems which are single-use, such as PLGA [14,16].

We believe that based on its molecular structure, SW033291 could be a compatible drug for CD platforms, and that the combination device (CD MPs, loaded with SW033291) will require less drug to achieve minimally effective concentrations and will reduce off-target effects in at-risk patient populations, ultimately improving patient outcomes in IPF. This is the first study to investigate the potential pairing of SW033291 with a cyclodextrin-based delivery system, and will hopefully lay the foundations for future studies investigating the efficacy of an SW033291/cyclodextrin combination product to extend drug delivery duration and localize SW033291’s physiological effects. Herein, we investigate and characterize the synthesis, loading, in vitro kinetics, and preliminary in vivo tolerance of CD MPs loaded with SW033291 in murine models to work towards a novel, sustained 15-PGDH inhibitor delivery system.

## 2. Materials and Methods

### 2.1. Materials

Soluble β-cyclodextrin (β-CD), lightly cross-linked with epichlorohydrin, referred to in this study as ‘prepolymer’, was purchased from CycloLab (Budapest, Hungary). Ethylene glycol diglycidyl ether (EGDE) crosslinker was purchased from Polysciences, Inc. (Warrington, PA, USA). (+)SW033291 was provided by Dr. Sanford Markowitz (Case Western Reserve University). The Lad2 cell line was provided by Dr. Dean Metcalfe (NIAID). Phosphate-buffered saline (1× PBS) was purchased from Millipore Sigma (Burlington, MA, USA). Corning transwell plates and all other reagents, solvents, and chemicals were purchased from Thermo Fisher Scientific (Hampton, NH, USA) in the highest grade available.

### 2.2. In Silico “Affinity” Predictions: Cyclodextrin and SW033291

SW033291 was determined to be an appropriate fit for CD delivery platforms through in silico analysis, utilizing both molecular docking software, PyRx v. 0.9.7, running the Autodock Vina algorithm (https://pyrx.sourceforge.io/, accessed on 7 October 2021, Released 2017, Molecular Graphics Laboratory, The Scripps Research Institute, La Jolla, CA, USA) and a cyclodextrin affinity predictor algorithm, developed by our group [17]. Molecular structure data files for SW033291 were downloaded from PubChem and cyclodextrin variants (α-CD, β-CD, and γ-CD) were isolated using Jmol v. 14 (http://www.jmol.org/, accessed on 7 October 2021) from Protein Data Bank entries 1CXF, 3CGT, and 1D3C, respectively. Previous studies have shown that “binding affinity”, represented by K_D_, is closely correlated with the duration of drug delivery, and that drug complexation with CD does not impact the chemical properties of the drug payload [11,18,19,20].

### 2.3. Cyclodextrin Microparticle Synthesis and Characterization

Similar to previously established protocols for synthesizing β-CD microparticles (β-CD MPs), the epichlorohydrin-crosslinked β-cyclodextrin prepolymer was solubilized in 0.2 M potassium hydroxide (25% *w*/*v*) and heated to 60 °C for 10 min [14,21,22,23]. Light mineral oil was warmed in a beaker with a Tween85/Span85 solution (24%/76%) and stirred at 500 rpm. Ethylene glycol diglycidyl ether (EDGE) was added dropwise, and the solution was vortexed for 2 min before adding the crosslinking solution to the oil/Span85/Tween85 mixture. During crosslinking (3 h), temperature and mixing speed were maintained at either ‘Method A’: 70 °C at 650 rpm or ‘Method B’: 60 °C at 1500 rpm.

The microparticles were then centrifuged at 200× *g* to be separated from the oil mixture and washed with excess hexanes twice, excess acetone twice, and finally, deionized water (diH2O) twice. The microparticles were resuspended in diH_2_O, frozen, and lyophilized for 72 h.

Particle size was determined by a Nikon Eclipse TE300 inverted microscope (Nikon Inc., Tokyo, Japan) and analyzed for particle diameter in ImageJ. Previous studies have cited particle sizes of 81.88 ± 36.86 µm with ‘Method A’ synthesis [22].

Some particle groups were also crushed with a mortar and pestle for 2–3 min—referred to as ‘crushed’—which is intended to reduce particle size after synthesis by ~70–80% [22].

### 2.4. In Vitro Reduction in Lad2-Generated 15-PGDH Due to SW033291-Loaded β-CD MPs

The in vitro activity of SW033291 loaded in β-CD MPs was assessed by treating the Lad2 cell line and assessing the enzyme inhibition activity, in a previously described assay [24,25]. Lad2 cells have previously been shown to highly express 15-hydroxyprostaglandin dehydrogenase (15-PGDH); therefore, they were chosen as a model cell-line for assessing PGDHi [24,26]. Briefly, either 1 mL of 600 µM SW033291 (25% DMSO in 2% FBS media) or 20 mg/mL loaded β-CD MPs (2% FBS media) were added to the top portion of the 0.4 µm pore Transwell plate. The bottoms of the plate were seeded with 200 k/mL LAD2 cells in 2 mL 2% FBS media and the plates were incubated at 37 °C in static conditions. At each time point, 1 mL of media was sampled from the bottom wells, 0.1 mL of solution was sampled from the top well and replaced with fresh media, and the top wells were transferred to a fresh plate. Enzyme activity from bottom media samples was recorded as counts per minute (CPM) over one hour and normalized to protein input mass (mg).

For each well, 20 mg/mL β-CD MPs were loaded for 24 h in 250 μM SW033291, washed twice with diH_2_O, and resuspended in media.

### 2.5. Liquid Chromatography/Mass Spectroscopy Procedure for SW033291-Loaded β-CD MPs Analysis

SW033291 concentrations in sampled aliquots were determined via LC-MS/MS in the Preclinical Pharmacology Core at UT Southwestern. Then, 0.1 mL of each sample was incubated at room temperature for 10 min with a twofold volume of acetonitrile, containing 0.1% formic acid and 50 ng/mL tolbutamide to induce solution crashing. Once precipitation occurred, samples were spun at 16,100× *g* for 5 min and the supernatant was transferred to a new tube and spun again. The supernatant was transferred to HPLC vials with inserts and analyzed by LC-MS/MS (Sciex 3200 QTRAP mass spectrometer coupled to a Shimadzu Prominence LC). An Agilent C18 XDB column (5 micron packing 50 × 4.6 mm) at 1.5 mL/min with solvents: (A) water +0.1% formic acid and (B) MeOH + 0.1% formic acid was used for chromatography prior to introduction of the sample into the mass spectrometer. Concentrations of SW033291 were determined by comparison with a standard curve made by spiking a blank matrix with varying concentrations of SW033291 which were processed as for samples.

### 2.6. Improvement of β-CD MPs Loading Protocol with SW033291

**Figure 1:**
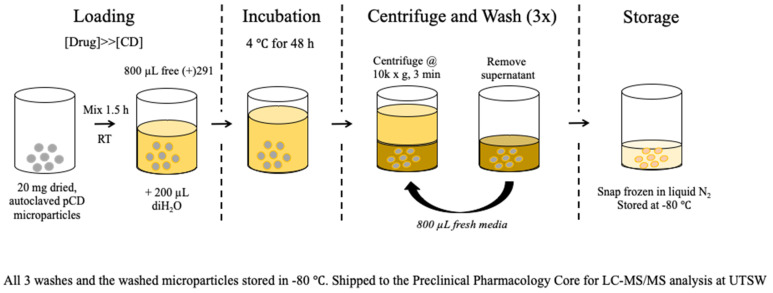
Loading protocol schematic for creating SW033291-loaded β-CD MPs. Prolonging loading to 72 h helps ensure the complexation of SW033291 within the cyclodextrin hydrophobic ‘pocket’.

A traditional drug loading procedure (single solvent, 250 μM drug, 24 h incubation) was found to only fill approximately 20–30% of available host CD molecules within the pCD MPs. To help drive the thermodynamic loading of SW033291 into β-CD MPs, 20 mg of dried MPs was mixed with 800 μL of 20 mg/mL SW033291 in DMSO for 1.5 h, followed by the addition of 200 μL diH_2_O mixed for 0.5 h. The addition of water is designed to drive the hydrophobic SW033291 into the MPs. The final 4:1 *v*/*v* organic:polar mixtures (final concentration of 16 mg/mL SW033291) were then incubated for at least 48 h at 4 °C. Microparticles were spun down at 10,000 rpm for 3 min and washed twice with 800 μL media/PBS (Figure 1).

### 2.7. Assessing β-CD MP Injectability

Larger diameter β-CD MPs have previously been shown to clog smaller-diameter needles. To ensure our synthesized MPs were injectable from a 29-gauge needle (BD, SafetyGlide Insulin Syringe), both the approximate settling time (seconds) and subjective injectability (binary: ‘yes’ or ‘no’) were obtained for β-CD MP formulations.

### 2.8. In Vivo Tolerance Murine Model

A murine model was used to test β-CD MP tolerance and quantify the extended delivery of SW033291 in vivo. Eight-week-old female C57/Bl6 mice were purchased from the Jackson lab (CWRU). SW033291-loaded β-CD MPs (2 mg, in 200 μL PBS) were administered retro-orbitally (RO) and peripheral blood was collected into Microtainer serum-separator tubes (Becton-Dickinson) by submandibular cheek puncture at designated time points (*n* = 4 mice per arm; 24, 72, 168, and 336 h post-administration). Whole blood was allowed to clot at room temperature and then spun at 6000× *g* for 3 min to separate the serum. Serum was removed and stored at −80 °C prior to sending samples to the Preclinical Pharmacology Core at UTSW for LC-MS/MS analysis. Analysis was conducted as described above, except the matrix utilized was mouse serum rather than culture media.

### 2.9. Statistical Analysis

Experiments were all carried out in triplicates, unless otherwise stated, and data are presented as the mean ± standard error. In vivo data represent the average of *n* = 4 mice, and error bars also represent the standard error of the mean.

## 3. Results

### 3.1. SW033291 Is Predicted to Have Significant Affinity for β-CD Host In Silico

**Figure 2:**
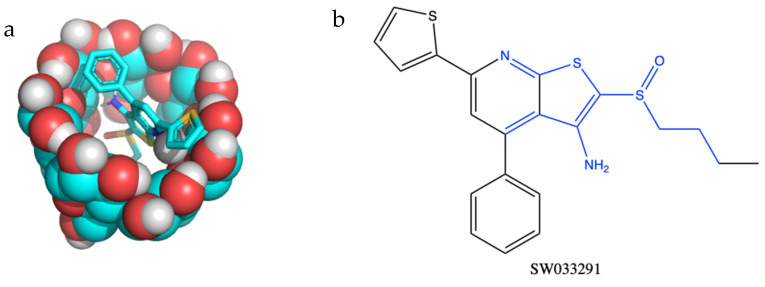
(**a**) Molecular structure in silico model demonstrating inclusion complex formation between the small-molecule drug SW033291 binding to the inner pocket of β-cyclodextrin. (**b**) Regions of SW033291 in blue are complexed within the cyclodextrin ‘pocket’.

Before beginning in vitro studies, we confirmed that a cyclodextrin delivery platform would be compatible with (+)SW033291’s chemical structure in silico. Utilizing two methods of affinity prediction—docking simulations and quantitative structure activity relationship (QSAR)—we found that SW033291 had the highest binding affinity with the β form of cyclodextrin (Table 1). Docking simulations also revealed that the central moiety which binds with cyclodextrin includes SW033291’s central heterocyclic rings and sulfoxide group (Figure 2). Based on previous studies, we predicted that a binding affinity of −23 KJ·mol^−1^ would result in a window of release of about 1–2 weeks [21,27,28,29].

### 3.2. Lowering the Temperature of Crosslinking and Increasing the Speed of Mixing Generates Smaller β-CD MPs

**Figure 3:**
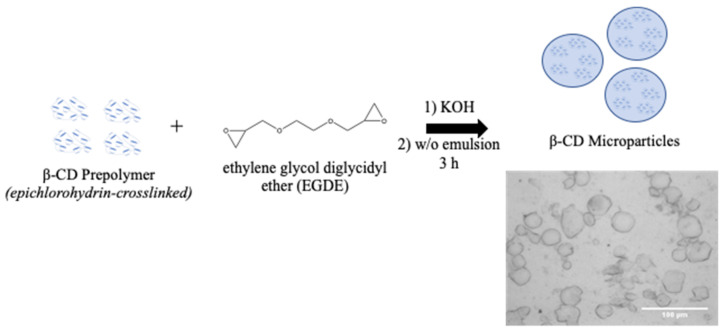
Overview of β-CD microparticle preparation. β-CD prepolymer is crosslinked with EDGE for 3 h at elevated temperature (60–70 °C) and formed in a water/oil (w/o) emulsion.

**Figure 4:**
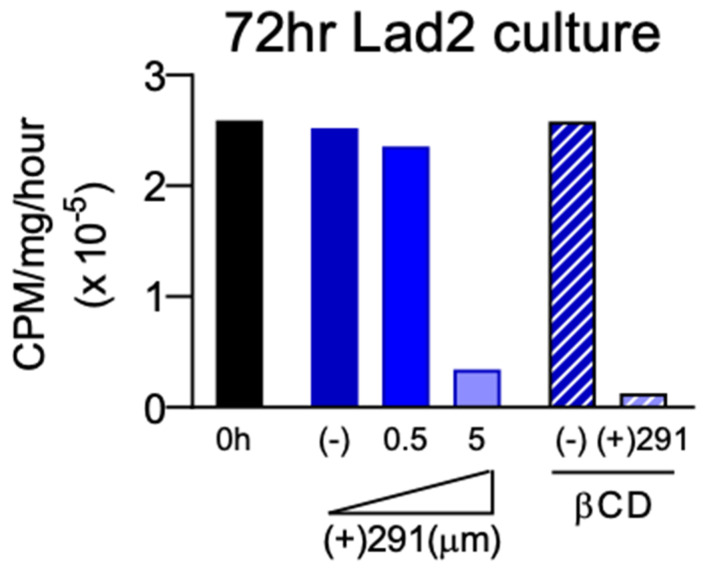
Incubation study of (+)SW033291-loaded β-CD MPs with Lad2 cells indicated sustained delivery in co-culture inhibits enzyme activity 72 h post-administration with similar efficacy as bolus SW033291 administration. (−) indicates the negative control group.

Established protocols for synthesizing β-CD MPs generate diameters that are not suitable for small-gauge needle injections; therefore, we sought to generate MPs with smaller diameters to allow injectability (Figure 3, Table 2). To this end, we modulated parameters during polymerization—specifically, temperature and mixing speed during polymerization—to generate smaller microparticles. We also explored manually crushing MPs with a mortar and pestle, which was previously reported to reduce particle diameters by 30% [22]. By reducing the temperature and raising the mixing speed to 1500 rpm, we generated MPs ~40 μm in diameter, a 50% reduction from the original protocol (Figure 4). Microparticles were then loaded for 24 h with (+)SW033291, and final loading concentrations were reported after drug leaching.

### 3.3. SW033291 Released from β-CD MPs Has Prolonged Lad2 Inactivation Compared to Free Drug

To confirm drug activity after delivery from β-CD MPs, (+)SW033291 activity was analyzed in vitro by proxy of analyzing supra-physiological concentrations of Lad2 enzyme activity inhibition. Using Method A, with uncrushed particles (final loading concentration: 11.9 mg/mL), we found that β-CD MP-released (+)SW033291 was effective at reducing LAD2 enzyme activity comparable to 5 μM free drug after 72 h (Figure 4). LAD2 controls and LAD2 treated with unloaded β-CD MP controls saw unremarkable changes in enzyme activity, confirming that β-CD alone does not contribute to enzyme inactivation. Drug release kinetics were also observed to exhibit increasingly zero-order kinetics compared to bolus administration, confirming that β-CD MPs slowed the rate of release of (+)SW033291 over a three-day period (Figure S2).

### 3.4. β-CD MP Loading Optimization

**Figure 5:**
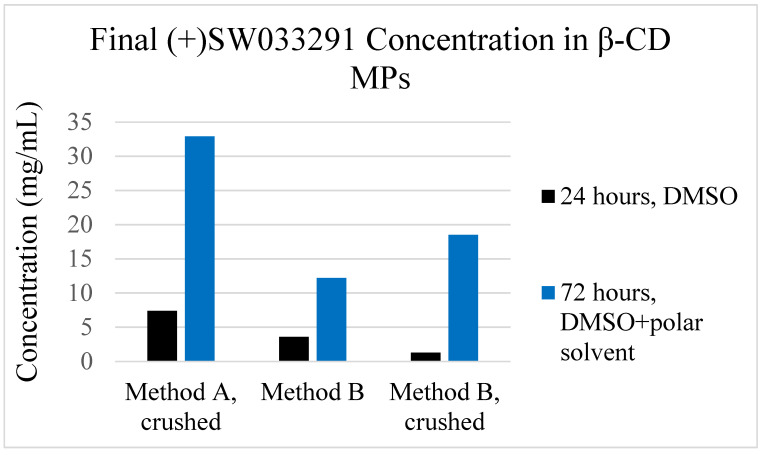
Final loading concentrations for β-CD MP formulations utilizing a ‘24-h’ loading protocol and our investigated ‘72-h’ loading protocol, which uses loading solution of both DMSO and a polar solvent (water) to help drive complexation (referred to as “DMSO + polar solvent”).

To help address the relatively low observed ‘final loading concentration’ seen in the 24 h loaded β-CD MP, we altered the loading protocol to encourage increased packing of drug within the microparticles. Comparing ‘24 h loading’, which used 24 h incubation in (+)SW033291 dissolved in DMSO, with ‘72 h loading’, which has previously been described as mixing a polar and nonpolar solvent to encourage complexation of the hydrophobic drug within the CD pockets. We found that altering this procedure yielded final loading concentrations 4- to 14-fold greater compared with our previous protocol (Figure 5).

### 3.5. At Lower Concentrations, β-CD MPs Are Injectable but Are Prone to Clumping

Before moving to an in vivo model, we aimed to investigate whether smaller-diameter β-CD MPs are suitable for injection from 29-Gauge syringes. In a serial dilution of 40 mg/mL, which was determined to be the maximum concentration of MPs achievable in an aqueous medium without immediate crashing, we found that the formulations were injectable ≤ 10 mg/mL; however, issues with clumping were observed (Table 3). At a lower concentration of ≤1.3 mg/mL, there was notably less clotting observed. Moving forward in vivo, we selected 10 mg/mL in order to ensure maximum β-CD MP delivery in a suitable volume (~200 μL).

### 3.6. (+)SW033291-Loaded β-CD MPs Prolonged Payload Delivery over 1 Week and Were Well Tolerated in a Murine Model

**Figure 6:**
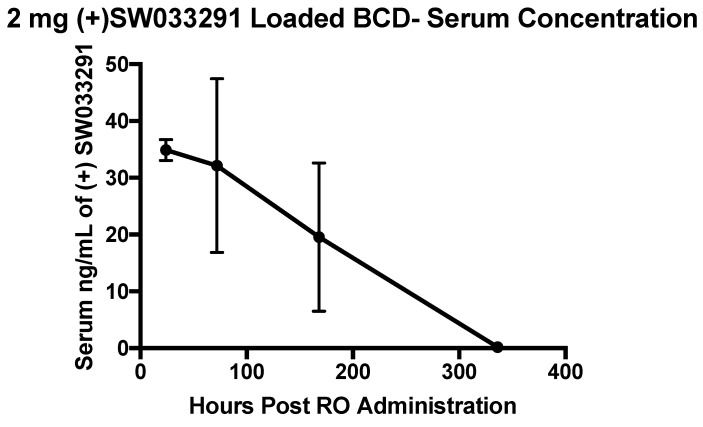
Serum (+)SW033291 concentrations collected from mice (*n* = 4 at each timepoint) following RO administration of (+)SW033291 loaded β-CD MPs. Error bars are representative of the standard error of the mean.

SW033291-loaded β-CD MPs (2 mg, in 200 μL PBS) were administered retro-orbitally (RO) to 8-week-old female C57/Bl6 mice (80 mg/kg) and peripheral blood was collected into Microtainer serum-separator tubes (Becton-Dickinson) by submandibular cheek puncture at designated time points (*n* = 4 mice per arm; 24, 72, 168, and 336 h post-administration). Within the cohort of 16 mice, we observed no adverse events following RO administration within the 2-week study. Serum drug concentrations (*n* = 4 at each timepoint) revealed that (+)SW033291 was still present after 1 week post-administration of SW033291 loaded β-CD MPs, eventually becoming undetectable in three out of four mice at the 2-week timepoint (Figure 6).

## 4. Discussions

Although pCD delivery systems have been used to extend the delivery of other small-molecule therapeutics, this is the first study showing that pCD is a compatible sustained release platform for PGDHi by (+)SW033291. Using in silico methods for affinity prediction, we found that SW033291’s structure was most compatible with β-CD (Figure 2). We found that (+)SW033291–β-CD interactions had a comparable free energy of binding to existing drugs that have been successfully delivered from pCD platforms (e.g., doxorubicin) (Figure S1). We then fine-tuned existing synthesis protocols for our lab’s β-CD MPs, generating a smaller-diameter microparticle of around 30–35 µm (Figure 3), confirming that decreasing the temperature and increasing the rate of stirring during crosslinking in a water/oil emulsion generates smaller pCD particles, helping to build on previous work [21,22].

Effective delivery of (+)SW033291 from β-CD MPs was also confirmed, first in vivo by effectively reducing the supra-physiological enzyme activity in LAD2 cells (Figure 4), and secondly in vivo in healthy mice (Figure 6). Drug kinetics in vitro revealed that (+)SW033291 was taking advantage of pCD inclusion, because the time of release was extended up to 6 days—compared with a 1-day bolus input—and exhibited increasingly zero-order kinetics (Figure S2).

(+)SW033291 loading protocols for β-CD MPs were also improved after discovering that 24 h incubation times in DMSO were insufficient to promote drug inclusion. To this end, we modified existing loading protocols by extending incubation time to 72 h and adding an aqueous component to the loading solution to help promote drug inclusion to pCD pockets (Figure 1 and Figure 5). This further confirms that drug inclusion within cyclodextrin and other similar drug delivery systems is highly dependent on solvent contact time and respective solubility of the drug within the drug-loading solvent [30,31,32].

It has previously been reported that cyclodextrin-derived polymers are relatively well tolerated in vivo; however, we found that 2 mg of β-CD MPs administered retro-orbitally was tolerated without ramifications in all mice within our cohort (Figure 6) [33]. We also observed that a single dose of (+)SW033291-loaded β-CD MPs was able to produce a drug delivery profile of over 1 week in vivo (Figure 6).

This study represents the groundwork for developing an extended drug delivery system for anti-fibrotic therapies. Although we were able to access global blood serum levels of (+)SW033291, future studies will focus on analyzing the biodistribution of the β-CD MPs and whether or not drug localization is achieved in regions of interest, specifically in the pulmonary tract and hepatic system [25]. The window of delivery that was achieved in vivo greatly surpassed bolus injection; however, the amount of drug released from 2 mg most likely would be subtherapeutic. Therefore, we propose to increase the frequency of β-CD MP administration in future models to help ensure that drug concentrations surpass minimally effective concentrations. We also aim to reduce MP clumping during administration, in which either administration protocols or synthesis quality (reducing polydispersity) can be changed to make MP injections easier [34]. In addition, altering the physical properties of the injection solution may allow for particles to remain in suspension for a longer time and might be better tolerated in vivo [35]. Ultimately, we aim to further optimize the pCD platform for the sustained delivery of (+)SW033291 and assess the efficacy of sustained PGDHi delivery as a therapeutic strategy in multiple models of chronic fibrotic disease.

## 5. Conclusions

Herein, we present the foundation for creating an injectable, cyclodextrin-based microparticle system for sustained 15-PGDH inhibition. As age-related fibrosis increases in prevalence, the long-term delivery of antifibrotic therapeutics will greatly improve upon current standards of care and augment the clinical usability of potent small-molecule fibrosis inhibitors, such as (+)SW033291. Extending the delivery of (+)SW033291 for over one week in vivo represents the first step in creating a novel tolerated strategy to reduce fibrotic deposition and improve morbidity and mortality in murine pulmonary and hepatic fibrosis. Future success in prolonged (+)SW033291 delivery would also have additional clinical implications for a number of fibrotic conditions.

## Figures and Tables

**Table 1:** Affinity binding simulations of 15-PGDH inhibitor SW033291 (CID:337839) complexation with α, β, and γ cyclodextrin (CD) in both PyRx and a machine learning algorithm for affinity prediction [17].

Ligand	Host	Binding Affinity—PyRx (KJ/mol)	Binding Affinity—ML Model (KJ/mol)
15-PGDH inhibitor SW033291	α-CD	−17.9 ± 1.7	−15.7
15-PGDH inhibitor SW033291	β-CD	−23.0 ± 1.6	−18.2
15-PGDH inhibitor SW033291	γ-CD	−22.0 ± 1.0	−14.3

**Table 2:** Microparticle batches synthesized with varying temperature and mixing speeds. Particles were then loaded with (+)SW033291 for 24 h (DMSO). Particle diameters were determined by ImageJ, and are presented as the mean ± S.D. Final loading concentrations were determined via LC/MS at UTSW.

Batch Preparation	Particle Diameter (µm)	Final Loading Concentration (mg/mL)
Method A, crushed	61.0 ± 26	32.9
Method B, uncrushed	41.2 ± 13	12.2
Method B, crushed	25.0 ± 9.5	18.0
Method B, crushed	35.0 ± 9.5	19.0

**Table 3:** Serial dilution of β-CD MPs in 1x PBS. Approximate settling times were measured subjectively when depositions of microparticles were first observed to crash out from solution. ‘Injectability’ was rated as a binary ‘yes’ or ‘no’ qualitative observation.

Concentration of β-CD MPs (mg/mL, d = 35 µm)	Approximate Settling Time (s)	Injectable? (29 G Needle)
40	5	no
20	9	no
10	16.6	yes
5.2	20	yes
2.6	23	yes
1.3	30	yes
0.7	>60	yes

## Data Availability

The data presented in this study are available within this article. Any additional requests for data will be promptly responded to by the corresponding author.

## Supplementary Materials

The following are available online at https://www.mdpi.com/article/10.3390/pharmaceutics14010085/s1, Figure S1: Affinity prediction machine learning model with training set (black) and highlighted drugs (“molecules of interest”) that were previously compatible with β-CD platforms. The dotted orange line is representative of SW033291’s predicted affinity to β-CD based on chemical descriptors. All chemical descriptor information for both host and ligand was sourced from PubChem, Figure S2: Drug release kinetics (n = 1) sampled from top transwell plates for both bolus-administered SW033291 and β-CD MPs loaded with SW033291 (incubated for 24 h). Trend lines represent a best linear fit, and trends with R^2^ values closer to 1 were considered “zero-order”.
