# Supplementary material for: Polymer Microparticles Prolong Delivery of the 15-PGDH Inhibitor SW033291
Source: Pharmaceutics. 2021 Dec 30;14(1):85. doi: 10.3390/pharmaceutics14010085 (PMC8779392; doi:10.3390/pharmaceutics14010085)
Supplement: Supplementary file 1 [file pharmaceutics-14-00085-s001.zip › pharmaceutics-1471129-supplementary.pdf]

pharmaceutics

# Supplementary Materials: Polymer Microparticles Prolong Delivery of the 15-PGDH Inhibitor SW033291

**Alan B. Dogan, Nathan A. Rohner, Julianne N. P. Smith, Jessica A. Kilgore, Noelle S. Williams, Sanford D. Markowitz, Horst A. von Recum and Amar B. Desai**

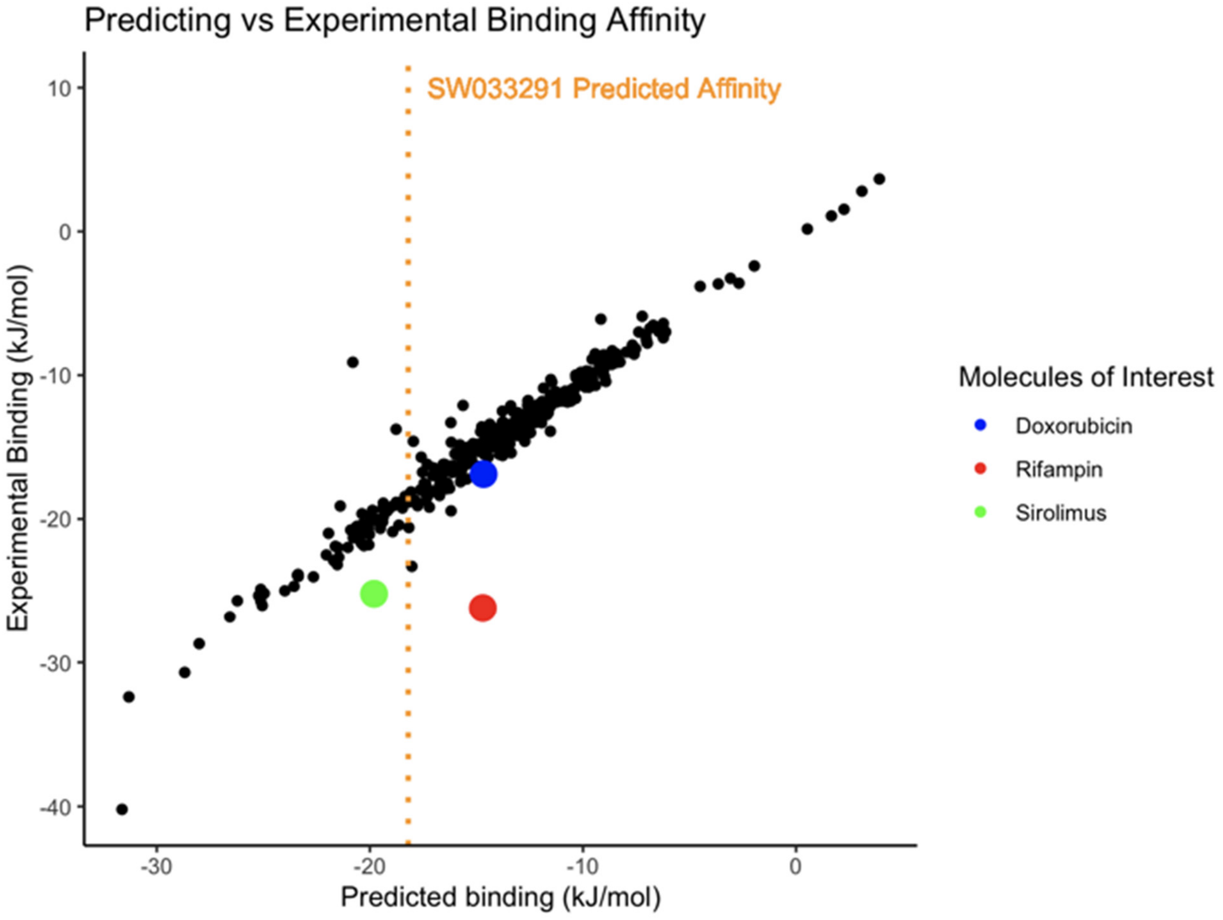


**Figure S1.** Affinity prediction machine learning model with training set (black) and highlighted drugs (“molecules of interest”) that were previously compatible with β-CD platforms. The dotted orange line is representative of SW033291’s predicted affinity to β-CD based on chemical descriptors. All chemical descriptor information for both host and ligand was sourced from PubChem.

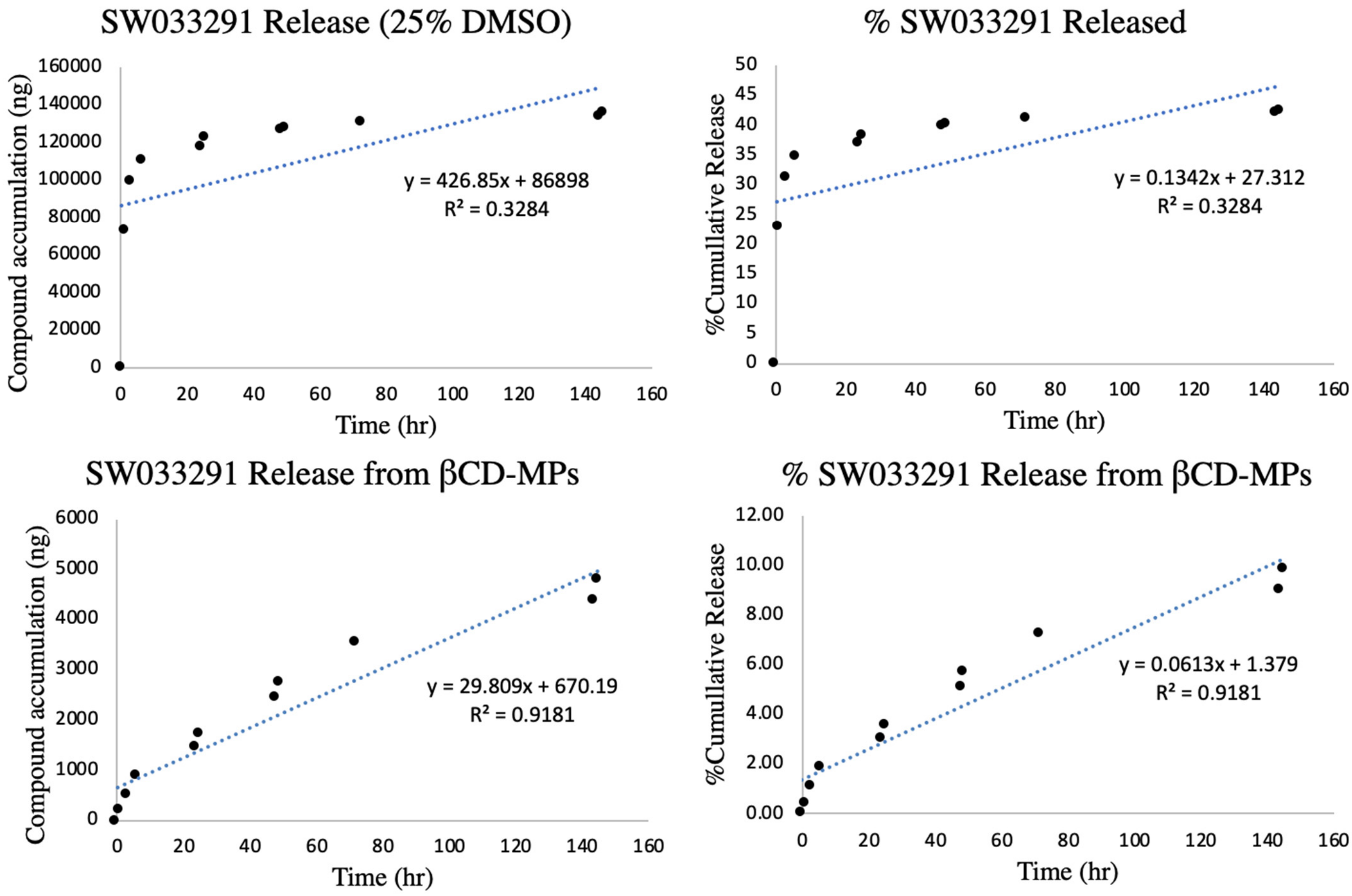


**Figure S2.** Drug release kinetics (n=1) sampled from top transwell plates for both bolus-administered SW033291 and β-CD MPs loaded with SW033291 (incubated for 24 h). Trend lines represent a best linear fit, and trends with $R^2$ values closer to 1 were considered "zero-order".
